# Does kinesiotaping can improve static stability of the knee after anterior cruciate ligament rupture? A randomized single-blind, placebo‐controlled trial

**DOI:** 10.1186/s13102-021-00248-6

**Published:** 2021-03-16

**Authors:** Katarzyna Ogrodzka-Ciechanowicz, Grzegorz Głąb, Jakub Ślusarski, Artur Gądek, Jolanta Nawara

**Affiliations:** 1 Institute of Clinical Rehabilitation, Faculty of Motor Rehabilitation, Institute of Physical Rehabilitation, University of Physical Education, Al. Jana Pawla II 78, 31-571 Krakow, Poland; 2 Department of Orthopedics and Traumatology, University Hospital, Krakow, Poland; 3 Department of Orthopaedics and Physiotherapy, Jagiellonian University Collegium Medicum, Kraków, Poland; 4 Liesing Orthopedic Center, Vienna, Austria

**Keywords:** Kinesio Tape, Anterior Cruciate Ligament Injuries, Static Stability, Postural Control

## Abstract

**Background:**

The aim of the study was the assessment of the early impact of the selected kinesiotaping technique on the static stability of the knee joint in patients with ACL rupture on the basis of stabilographic parameters.

**Methods:**

Sixty-two patients with a complete ACL rupture (32 patients in experimental group and 30 patients in placebo group) took part in the randomized single-blind, placebo-controlled trial. The ligament technique of KT was taken into consideration. Application of a KT tape only on the injured knee was to stabilize the knee joint. Experimental group had application of KT on the injured knee and the placebo group had a KT placebo application (with no tension on KT). Intervention and stabilographic test in both groups was the same.

Research tools included measurements of static stabilographic parameters on stabilometric platform CQStab2P®. Outcome measures were assessed before intervention and after KT application. The analysis included evaluation of outcome variables – total path length, (SP), statokinesiogram path length in the XY axes (SPML, SPAP), and mean velocities in the XY axes (MV, MVML, MVAP).

**Results:**

The results show a statistically significant shortening of the SP, SPAP and SPML variables only in experimental group. In the placebo group the results were not significant. The analysis also showed a significant improvement in all analyzed variables in the experimental group compared to the healthy side. In the placebo group, the results did not improve significantly after KT application compared to the healthy side.

**Conclusions:**

Application o

f KT in patients after ACL rupture shortened the total path length and improved the value of parameters in the frontal and sagittal planes in experimental group, which may suggest the potentially greater improvement in these parameters. By improving the values of the analyzed variables, the KT application is able to compensate for the loss of static stability of the knee.

**Trial Registration:**

This study was registered retrospectively in the Australian New Zealand Clinical Trials Registry (ANZCTR). Registration number: ACTRN12616001407482.

## Background

To maintain proper postural control of the human body, it is necessary to maintain a balance between proprioception, joint mobility and neuromuscular control [1, 2]. Stability in standing and walking is controlled by musculoskeletal, somatosensory, and vestibular systems [3].

The anterior cruciate ligament (ACL) rupture results in disorders of its function, which in turn affect the changes in the biomechanics of the body [4].

As a result of a failure of ACL function due to an injury, there is loss of knee stability. In particular, anteromedial stability is impaired, which leads to significant deterioration of a patient’s ability to maintain a standing position and control of their centre of gravity [4].

It should be noted that knee dysfunction due to ACL rupture is not only related to the loss of mechanical stabilisation. During the injury, there is also impairment of the receptors located within the ligament which are necessary for proper functioning of joint proprioception. In addition, it is assumed that sensory disorders may significantly affect the occurrence of abnormalities in posture control [5].

Moreover, in patients with the ACL rupture there have been reported disorders of functioning of the muscles of the neck, head and trunk, leading to abnormal postural control. The stabilographic examination of these patients showed a significant anterior shift of the centre of pressure of the foot (CoP) in the sagittal plane and the shift towards the side in the frontal plane. It should therefore be stated that the ACL injury leads to impairment of its own function and, as a consequence, the biomechanics within the entire body is disturbed [6].

Negahban et al. after analyzing 12 studies clearly indicates that in patients with isolated ACL injury postural control is impaired in both legs, especially injured leg. The result of within-group difference in eyes open condition confirms bilateral deficit of postural control [7].

Kinesiotaping (KT) is a therapeutic method, supporting the process of rehabilitation in many diseases and injuries of the musculoskeletal system. It consists in sticking selected fragments of the body with special structure tapes. It directly affects the skin (mainly Ruffini’s ends, receptors of pain, deep sensation), musculoskeletal and lymphatic system [8, 9].

Kinesiotaping method originates from Japan, where in the early 70’s a Japanese chiropractic, the president of the Kinesiotaping Society, Dr. Kenzo Kase, began working on a new therapeutic method. He was the first to apply kinesiotaping in the treatment of joint disorders and rheumatism. In Europe, kinesiotaping has been used since 1996. Initially, kinesiotaping was mainly used in sport as a prevention of injuries and as a therapy supporting the treatment of injuries. Currently, it applies to practically every muscle and joint dysfunction. The purpose of the method is to use natural self-healing processes. KT uses special kinesio tapes, which are characterized by flexibility and extensibility only to the length, thanks to which it is possible to apply it to any part of the body with different tension, used depending on the type of application and the therapeutic result we want to achieve. The therapeutic effect of the tape is associated only with the appropriate technique of application. KT reduces muscle and joint pain, supports muscles and joints, corrects joint settings and removes lymphatic edema. The advantage of the method is primarily the fact that this technique does not limit joint movements and is not an obstacle to running the current lifestyle [10–12].

KT is the recommended and used method to improve coordination during standing and walking. KT is widely used in orthopedic, traumatic and neurological patients because it successfully improves static stability. Two theories can be found in the literature that describe the influence of KT on stability. The first theory assumes that KT significantly affects the structural stiffness of the joints, and thus constitutes a postural control mechanism. The second theory, on the other hand, describes the effect of KT on skin extensibility, which causes irritation of proprioreceptors and the transmission of sensory information to the central nervous system. The information from the sensory receptors improves the control of standing and posturę [3].

The literature describes widely the use of KT in the treatment of patients with many dysfunctions, as well as is used in the prevention and support of sports injury treatment [9, 10].

But in the literature there is no clear answer favouring the efficiency of KT in several motor function disorders. In many cases application of KT has brought expected results, on the other hand, many applications have not showed positive effects [13–15].

The use of stabilizing and muscle applications in improving muscle balance and strength in ankle injuries in athletes is described. Controversy arouses both the stabilization of the joint in the context of the obtained results of high-speed and proprioceptive action [16].

There are also many studies confirming the effectiveness of various KT applications in reducing inflammation and pain by increasing the flow of lymph and blood or increasing the range of motion [17–19].

In addition, the application of kinesiotaping in a patients who have been diagnosed with significant weakness of the quadriceps has resulted in a significant improvement in efficiency of the lateral and medial head of the quadriceps. Increasing the activity of these muscles may have a positive influence on improving the efficiency of the knee [20].

Considering the changes in postural control in patients with ACL rupture and the properties of KT, it is therefore possible to use KT in improving standing stability?

Taking into account the above abnormalities in the body biomechanics in patients with ACL rupture, the possibility of application of the KT method in therapy and up to date, to the best of our knowledge there is, however no research on this subject, own research will present possible use of kinesiotaping for correction of changes in the motor system of patients with instability of the knee.

The aim of the study was to assess the early impact of the selected KT technique on the stability of the knee joint during standing position in patients with ACL rupture based on stabilographic parameters.

This study is based on the hypothesis that KT improves static stability of the knee joint in patients with ACL rupture during standing position.

## Methods

### Design

This pre-posttest repeated measure randomized single-blind, placebo-controlled trial was reported according to the recommendations of the CONSORT statement [21].

The first author (physiotherapist) enrolled 70 patients with ACL rupture. Those who qualified for the research were patients at the Department of Orthopaedics and Rehabilitation of University Hospital in Krakow.

Before starting the intervention, patients were randomly allocated to the experimental or placebo group by an independent researcher using the sealed envelopes method. Patients from placebo group were blinded after assignment to intervention.

Eligibility criteria for the research:

1. ACL rupture confirmed by imaging examination: MRI, CT SCAN.
2. The presence of no other injuries or illnesses which may affect the outcome of the tests (tests performed by an orthopedist).
3. After becoming acquainted with the purposes and the course of the research – a voluntary consent to participate in the study (written consent).
4. The patients had not taken medications which may affect motor coordination.

Exclusion criteria:

1. Injuries or illnesses which may affect the results (i.e. MCL/LCL/PCL injury, meniscus injury, degenerative changes in the joints of the leg).
2. Acute instability – surgery scheduled within one month from the injury.
3. Lack of patient’s consent to participate in the research.

The examination was performed before ACL reconstruction and none of the patients had yet undergone preparation for the reconstruction in the form of preoperative rehabilitation, so it had no effect on the effect of KT.

The study lasted from 2015 to 2018 and was carried out at the University of Physical Education in Krakow in Laboratory of the Diagnostics of the Motor System, Laboratory of the Motion Analysis.

### Intervention

During this research ligament technique of kinesiotaping (which limiting the anterior translation of the tibia) was taken into consideration. Application of a KT tape only on the injured knee was to stabilize the knee joint. One strip of an “I-type” KT was used. During the application, the patient was lying supine. The knee was flexed to 45° (measured by goniometer).

The base of the measured KT piece was applied without tension at the height of the patella tendon. Then the tape with a tension of 75 % was applied symmetrically on the medial and lateral sides of the knee up to the femur. In the ligament technique, the stretching of the tape oscillates between 75 and 100 %, because the maximum or submaximal extension of the tape supports passive joint stabilizers, which are unable to properly perform their function due to injury, in addition only such a force is able to maintain the dorsal glide [22, 23]. In own research, it was decided that all patients should have the same stretch of the tape, which amounted to 75 %, because a 100 % tension could cause damage to the skin during movement, as well as KT could peel off.

**Fig. 1:**
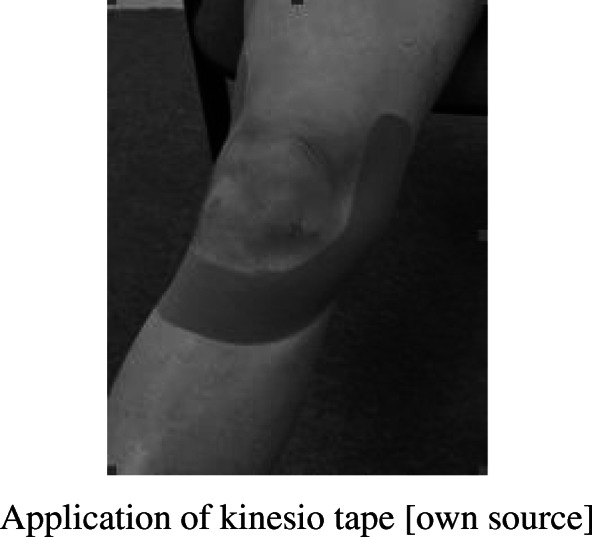
Application of kinesio tape [own source]

During the application, the patient was asked to extend the knee while the therapist applied the tape in the cranial-dorsal direction. The last 5 cm of tape were applied without tension on both the lateral and medial sides. (Fig. 1.)

The placebo group had a KT placebo application (with no tension on KT). Intervention and stabilographic test in the placebo group was the same as in the experimental group.

Analyses of results of injured leg for both groups were conducted twice:

1. at baseline – before application of KT,
2. after the application of KT.

After the application of kinesiotaping each patient had 1 hour to adjust to the application. This allowed early assessment of impact of the KT.

There were 13 patients with the dominant injured limb and 19 with the undominant injured limb in the Experimental group and 18 patients with the dominant injured limb and 12 patients with the undominant injiured limb in Placebo group.

### Outcome measures

The study used measurements of static stabilographic indicators with a stabilographic platform (*primary outcomes)* [24–26].

To assess stabilographic indicators a stabilographic platform CQStab2P® (Czernica, Poland) was used. CQStab software was used to archive and process results [27, 28].

The analysis of the course of the statokinesiogram line shows that for the accuracy of its reproduction (accuracy 1 mm with a fluctuation radius of 10 cm) the accuracy is 1 %. The 12 bit processing used (effective 10 bit) means the processing accuracy is 0.1 %.

Registration of the data was possible due to tensometric sensors placed on the surface of the platform. These sensors reacted to changes in pressure load of the patient’s feet on the ground, by registering the displacement of the centre of pressure of the foot on the ground (CoP). The results of the study were archived by charting changes in the position of CoP.

In the analysis of the results the following indices were used:

*SP* – total path length, on both axes of rectangular coordinate Y0X [mm],

*SPAP* – statokinesiogram path length on the Y axis [mm],

*SPML* – statokinesiogram path length on the X axis [mm].

MV– mean velocity of the COP in XY (2D) axes [mm/s].

MVAP– mean velocity of the CoP in the Y axis [mm/s].

MVML– mean velocity of the CoP in the X axis [mm/s].

Each of the patients had the opportunity to test the position on the platform just before the actual measurements. This “pre-test” lasted about 15 minutes and was not included in the actual test.

The first test was done after the patient had rested in a sitting position for 5 minutes. The patient was standing freely, barefoot, with upper limbs placed along the body and straight legs. During the measurement, subjects had to focus attention on a graphic point placed at eye level, within 2 m from the platform. The patient was then asked to stand on injured leg and flex the other knee to such extent that touching the ground was impossible, without changing the settings of the hip. Patient could not connect lower limbs, support the elevated leg on the currently examined leg. The study was carried in silence, with natural daylight. Each measurement was made at the same time of the day.

**Fig. 2:**
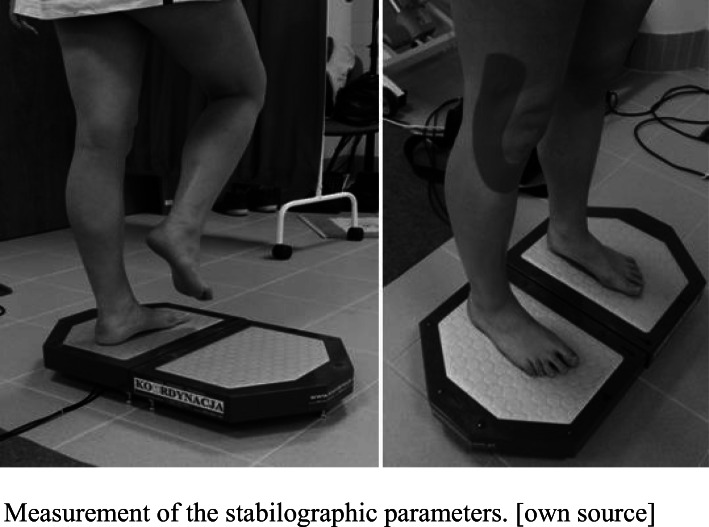
Stabilographic test [own source]

During the first examination four 30-second measurements were made (2 for each limb):

- Standing on the non-injured leg with eyes open (the opposite leg flexed in the knee).
- Standing on the injured leg with eyes open (the opposite leg flexed in the knee). (Fig. 2)

The results represent the averaged results from all measurements.

Then a physiotherapist applied KT. After the application of the tape and 1-hour break (with a 5-minute rest in a sitting position before the test) the second test was done. The test covered the same measurement as during test 1. Duration of intervention was about 1 hour and 30 minutes.

The study also included the eye-closed test, but over 50 % of patients did not complete the measurement, so only the results of the open-eye test were used in the analysis.

The results were compared with the control group.

The intervention was supervised by a physiotherapist (first author) who assessed whether the research was consistent with the methodology of the stabilographic examination and whether the KT application met the methodological assumptions.

### Statistical analysis

The STATISTICA 12.0.PL software was used for statistical analysis. The first stage of the analysis was to check the normality of the distribution of variables using the Shapiro-Wilk test. The next step was to determine the significance of changes between the variables. Two-way ANOVA analysis (ANOVA group x time) was used. Multiple comparisons were based on the Bonferroni correction. Cohen’s d allowed for the assessment of the effect size, which was analyzed in accordance with previous studies [29–31]. The study assumed *p* < 0.05. The paired t-test power analysis showed that at least 30 subjects were needed to obtain a power of 0.8 at a two-sided level of 0.05 at an effect size of d = 0.6.

## Results

**Fig. 3:**
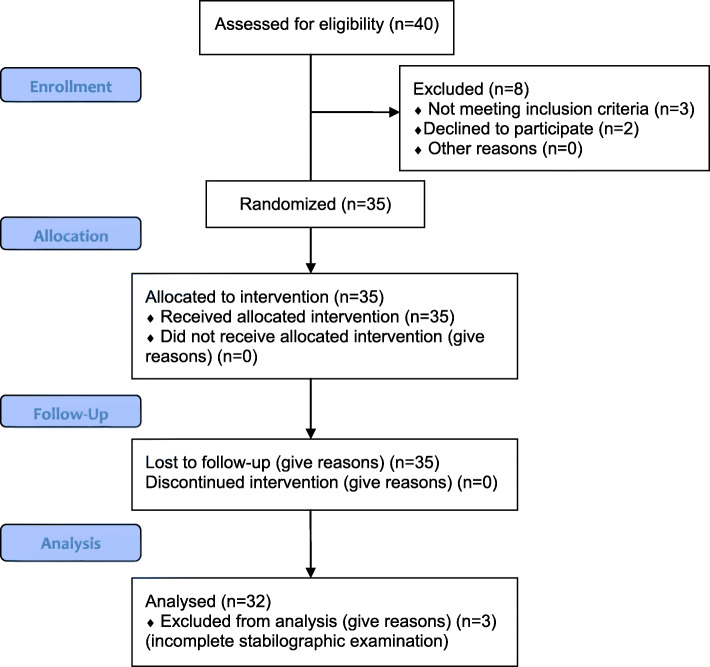
CONSORT Flow Diagram

70 patients were enrolled to the research project. After randomization 33 patients were qualified to the experimental group and 32 to placebo group. The final experimental group consisted of 32 patients (20 men and 12 women) aged 20–57 years (29.8 ± 9.5) and placebo group comprised a group of 30 patients (23 men and 7 women) aged 20–55 years (28.16 ± 6.17). (Table 1.) 5 patients were excluded in the research process (not meeting inclusion criteria (n = 3), declined to participate (n = 2). Figure 3. shows the qualification process for clinical trials.

All patients had been diagnosed (by an orthopaedist) with a complete ACL rupture within the knee joint (the average time between the injury and examination was 4 months +/- 2.4).

**Table 1:** Anthropometric data of the research group

Variable	Experimental		Placebo		*p*
	**x ± SD [yrs]**	**Min-Max**	**x ± SD [yrs]**	**Min-Max**	
**Age**	29.8 ± 9.5	20–57	28.16 ± 6.17	20–55	0.254
**Height [cm]**	175.1 ± 8.3	158.5–193	177.6 ± 8.6	160.5–189	0.329
**BMI**	25.3 ± 3.2	19.2–31.9	24.6 ± 4.4	22.8–28.4	0.254
**time between the injury and examination [mths]**	4+/-2.4				

*Experimental* experimental group; *Placebo* placebo group

***p*** < 0,05

Analysis of the SP indicator results before and after the application of KT in the experimental group indicates a significant improvement (*p* < 0.001) over the placebo group (*p* < 0.060).

Statistical analysis in experimental group showed a significant shortening (*p* < 0.001) of SPAP path while standing one-legged on the injured limb before and after application of KT.

After the application of KT, the results between the groups were statistically significantly different (*p* = 0.039). Improvement occurred in the experimental group.

Differences in measurements of the SPML variable before and after application of KT showed a statistically significant decrease in the SPML variable in experimental group. After KT application, the difference in the SPML values between the groups was statistically significant (p = 0.046) (Table 2.)

The analysis of the CoP velocity in the XY axes (MV) indicates a significant improvement in the results in the experimental group (p = 0.039). The comparison between the groups also indicates a significantly better result of the experimental group (p = 0.042) after the application of KT. The results of the CoP velocity in the Y axis (MVAP) and X (MVML) also indicate significant improvements after KT application in the experimental group and between the groups.

**Table 2:** Comparsion of outcome variables characterizing the measurement of the SP, SPAP variable at baseline and after application of KT

		Baseline	Post	X difference (95% CI)	***p***^***a***^	ES^**a**^
		X±SD	X±SD			
**SP [mm]**	**Experimental**	1107.3**±**288.8	927.8**±**295.9	-179.5 (1.81 to 2.01)	**<0.001**	0.61
	**Placebo**	1073.9**±**266.5	1033.1**±**289.2	-40.8 (1.45 to 2.56)	0.060	0.14
	***p***^***b***^	0.638	**0.043**			
	**ES**^**b**^	0.11	0.36			
**SPAP [mm]**	**Experimental**	818.3**±**238.4	622.4**±**254.8	-195.9 (2.05 to 2.91)	**<0.001**	0.79
	**Placebo**	702.4**±**219.3	698.1**±**217.5	-4.3 (1.01 to 3.01)	0.067	0.02
	***p***^***b***^	0.464	**0.039**			
	**ES**^**b**^	0.50	0.32			
**SPML [mm]**	**Experimental**	763.9**±**238.6	681.2**±**234.0	-82.7(2.21 to 3.34)	**0.031**	0.35
	**Placebo**	752.1**±**208.6	728.6**±**194.8	-63.5 (1.26 to 2.69)	0.221	0.11
	***p***^***b***^	0.882	**0.046**			
	**ES**^**b**^	0.05	0.22			
**MV** **[mm/s]**	**Experimental**	36.7±8.7	32.2±9.3	-4.5 (1.98 to 2.78)	**0.039**	0.49
	**Placebo**	37.3±±9.9	35.6±9.5	-1.7 (1.45 to 2.21)	0.089	0.17
	***p***^***b***^	0.079	**0.042**			
	**ES**^**b**^	0.06	0.36			
**MVAP** **[mm/s]**	**Experimental**	23.2±5.8	19.2±7.6	-4 (1.67 to 2.56)	**0.043**	0.59
	**Placebo**	23.8±7.4	22.2±7.9	-1.6 (1.75 to 3.01)	0.079	0.20
	***p***^***b***^	0.096	**0.039**			
	**ES**^**b**^	0.09	0.38			
**MVML** **[mm/s]**	**Experimental**	24.4±6.8	20.3±6.5	-4.1 (1.21 to 2.45)	**0.031**	0.61
	**Placebo**	25.1±6.4	23.1±6.2	-2 (0.92 to 3.42)	0.112	0.31
	***p***^***b***^	0.065	**0.024**			
	**ES**^**b**^	0.10	0.44			

Baseline the measurement before application of KT; Post the measurement after application of KT; *SP*the total path length; *SPAP* the path length on the Y axis; *SPML* the path length on the X axis; *MV* mean velocity of the COP in XY axes; *MVAP* mean velocity of the CoP in the Y axis; *MVML* mean velocity of the CoP in the X axis; *p*^*a*^*p*-value; between baseline and post-KT within each group; *p*^*b*^*p*-value between study groups; *ES*^*a*^ effect size (Cohen d) within each group; ES^b^ effect size (Cohen d) between study groups; *p* < 0,05; CI confidence interval

The results of the variables between the injured side and the healthy side in both groups were also compared.

In the experimental group, before the KT application, the results of all indicators differed significantly between the sides. After the KT application, the values of the injured side approached the results of the healthy side, and the differences were not statistically significant. (Table 3.)

There were statistically significant differences between sides in both the Baseline and second measurement in all of the analysed variables in Placebo group. (Table 4.)

**Table 3:** Comparsion of outcome variables characterizing the measurement of the SP, SPAP variable at baseline and after application of KT between injured and healthy side in Experimental group

		Baseline	Post	X difference (95% CI)	***p***^***a***^	ES^**a**^
		X±SD	X±SD			
**SP** **[mm]**	**Experimental** **injured**	1107.3**±**288.8	927.8**±**295.9	-179.5 (1.81 to 2.01)	**<0.001**	0.61
	**Experimental** **healthy**	945.3±319.47	935.8±347.9	-9.5 (1.34 to 1.56)	0.308	0.02
	***p***^***b***^	**0.043**	0.132			
	**ES**^**b**^	0.53	0.02			
**SPAP** **[mm]**	**Experimental** **injured**	818.3**±**238.4	622.4**±**254.8	-195.9 (2.05 to 2.91)	**<0.001**	0.79
	**Experimental** **healthy**	644.8±394.9	628.9±337.1	-15.9 (1.76 to 1.97)	0.478	0.04
	***p***^***b***^	**0.032**	0.254			
	**ES**^**b**^	0.53	0.02			
**SPML** **[mm]**	**Experimental** **injured**	763.9**±**238.6	681.2**±**234.0	-82.7 (2.21 to 3.34)	**0.031**	0.35
	**Experimental** **healthy**	690.4±276.3	683.8±230.2	-6,6 (1.53 to 1.79)	0.222	0.02
	***p***^***b***^	**0.045**	0.632			
	**ES**^**b**^	0.28	0.01			
**MV** **[mm/s]**	**Experimental** **injured**	36.7±8.7	32.2±9.3	-4.5 (1.11 to 1.43)	**0.039**	0.49
	**Experimental** **healthy**	32.8±6.2	31.7±5.9	1.1 (1.54 to 2.01)	0.121	0.18
	***p***^***b***^	**0.041**	0.179			
	**ES**^**b**^	0.51	0.06			
**MVAP** **[mm/s]**	**Experimental** **injured**	23.2±5.8	19.2±7.6	-4 (1.23 to 3.12)	**0.043**	0.59
	**Experimental** **healthy**	18.8±4.1	19.5±4.8	0.7 (0.23 to 1.95)	0.097	0.15
	***p***^***b***^	**0.036**	0.213			
	**ES**^**b**^	0.87	0.04			
**MVML** **[mm/s]**	**Experimental** **injured**	24.4±6.8	20.3±6.5	-4.1 (0.34 to 2.47)	**0.031**	0.61
	**Experimental** **healthy**	19.6±5.9	19.1±4.8	-0.5 (0.12 to 2.11)	0.173	0.09
	***p***^***b***^	**0.046**	0.095			
	**ES**^**b**^	0.75	0.21			

Experimental injured – injured side; Experimental healthy – healthy side

**Table 4:** Comparsion of outcome variables characterizing the measurement of the SP, SPAP variable at baseline and after application of KT between injured and healthy side in Placebo group

		Baseline	Post	X difference (95 % CI)	*p*^*a*^	ES^a^
		**X ± SD**	**X ± SD**			
**SP** **[mm]**	**Placebo** **injured**	1073.9 **±** 266.5	1033.1 **±** 289.2	-40.8 (1.45 to 2.56)	0.060	0.14
	**Placebo** **healthy**	967.3 ± 219.77	934.8 ± 285.1	-32.5 (1.24 to 1.64)	0.104	0.12
	***p***^***b***^	**0.049**	**0.045**			
	**ES**^**b**^	0.43	0.34			
**SPAP** **[mm]**	**Placebo** **injured**	702.4 **±** 219.3	698.1 **±** 217.5	-4.3 (1.01 to 3.01)	0.067	0.01
	**Placebo** **healthy**	625.8 ± 234.1	616.9 ± 237.6	-8.9 (1.32 to 1.84)	0.269	0.03
	***p***^***b***^	**0.027**	**0.018**			
	**ES**^**b**^	0.33	0.35			
**SPML** **[mm]**	**Placebo** **injured**	752.1 **±** 208.6	728.6 **±** 194.8	-63.5 (1.26 to 2.69)	0.221	0.11
	**Placebo** **healthy**	690.4 ± 276.3	683.9 ± 230.2	-6,5 (1.23 to 1.54)	0.138	0.02
	***p***^***b***^	**0.038**	**0.011**			
	**ES**^**b**^	0.25	0.23			
**MV** **[mm/s]**	**Placebo** **injured**	37.3 ± 9.9	35.6 ± 9.5	-1.7 (1.11 to 2.34)	0.089	0.17
	**Placebo** **healthy**	32.4 ± 5.6	31.8 ± 4.7	-0.6 (0.97 to 2.45)	0.076	0.11
	***p***^***b***^	**0.043**	**0.047**			
	**ES**^**b**^	0.60	0.50			
**MVAP** **[mm/s]**	**Placebo** **injured**	23.8 ± 7.4	22.2 ± 7.9	-1.6 (1.22 to 2.62)	0.079	0.20
	**Placebo** **healthy**	19.5 ± 5.9	18.9 ± 3.8	-0.6 (0.21 to 1.31)	0.139	0.12
	***p***^***b***^	**0.039**	**0.042**			
	**ES**^**b**^	0.64	0.53			
**MVML** **[mm/s]**	**Placebo** **injured**	25.1 ± 6.4	23.1 ± 6.2	-2 (1.29 to 2.97)	0.112	0.31
	**Placebo** **healthy**	19.9 ± 3.9	19.5 ± 4.6	-0.4 (0.34 to 2.49)	0.177	0.10
	***p***^***b***^	**0.047**	**0.034**			
	**ES**^**b**^	0.98	0.69			

Placebo injured – injured side; Placebo healthy – healthy side

## Discussion

Analysing the available literature, there is no information regarding the effectiveness of KT in the treatment of patients after a complete ACL ruptures. So far, no studies have been performed with the use of KT in improving the static stability of knee after one of the most frequent ligament injuries in the knee joint. Therefore, in this study the attempt was made to evaluate the effectiveness of selected ligamentous application of KT in cases of a complete ACL rupture. The aim of the study was to assess of the early impact of the selected KT technique on the static stability of the knee.

The presented research focuses on the evaluation of knee stability during standing position, evaluated by stabilographic platform.

Application of KT affected changes in the SPAP and SPML variables only in experimental group. The results show a statistically significant shortening of the statokinesiogram path length on the Y and X axes after application of KT in experimental group. It means that in terms of SPAP and SPML indicators, the KT application significantly improves static stability of the knee. The fact that the SPAP and SPML variables decreased after the KT application may indicate that the KT application causes a stimulation of mechanoreceptors, which enhances proprioception, joint position sense, and perception to avoid excessive movement.

The results of the SP variable were also analyzed. The results show a significant improvement in static stability in the experimental group after KT application. In the placebo group the result was not significant. It should therefore be concluded that the application KT reduces the value of sway at the CoP in terms of SP indicator.

The analysis of the results between the injured side and the healthy side in experimental group shows statistically significant differences. After KT application, the results improved significantly and came closer to the results of the healthy side. In the placebo group, both before and after the KT application, the indicators values differed significantly, which means that KT did not shorten the SP, SPAP and SPML pathways.

Based on the average COP velocity the nature of the balance dynamics can be defined. Low values of average velocity indicate a low dynamics of body balance control, so it can be assumed that the examined person is standing still. High values are evidence of the restless and sudden body movements [32].

Measurement of the CoP velocity in individual axes indicates a significant improvement of these parameters in the experimental group after the KT application. The reduction of velocity of the CoP deflection after KT application means that kinesiotaping decreased the CoP deflections, and thus the standing position was more stable.

The results suggest that the application of KT in participants with ACL rupture restores the SP, SPAP, SPML, MV, MVAP, MVAP indicators compared with the healthy side.

This may be due to the fact that thanks to the strong KT tension (75 %), the tape is able to provide stability to the joint, thus resulting in better control of the standing position, which reduces the CoP swaying.

In the research published to date, authors indicate the effectiveness of KT in the recovery of knee muscle strength in the course of the patellofemoral conflict or they state the usefulness of this method in the prevention of muscular injuries [12, 20, 33, 34]. According to Kalron et al. there are data concerning the effectiveness of this method in the acute phase of musculoskeletal injuries [18]. There are, however, no clear scientific reports on outcomes of KT, as well as studies assessing effectiveness of the therapy in most of the damage to the locomotor system among both patients and athletes. Similar conclusions were drawn by Mostafavifar et al. and Williams et al. [35, 36].

Kinesiotaping is also very often used in the prevention or treatment of sports injuries. However, also in this field of medicine, the results of research on its effectiveness are ambiguous.

Williams et al. analyzed 97 articles describing the effectiveness of KT in the treatment and prevention of sports injuries [36]. The results of their analysis indicate that the effectiveness of KT in the treatment of pain, improvement in muscle strength, range of motion and the impact on proprioception are ambiguous and controversial with small beneficial results. They recommend further experimental studies on the effectiveness of KT in sport..

Unfortunately, there are no studies on the effectiveness of KT in regaining or maintaining static stability of the knee. There are reports suggesting that the use of KT has a positive effect on proprioception in patients with an ACL rupture during gait. Therefore, the application may improve gait pattern as well as the improve subjective function of the affected knee joint [37]. Bischoff et al. made such conclusions only on the basis of gait analysis [37]. In turn, Liu et al. investigated whether KT application improves proprioception, balance, and functional performance in patients with ACL rupture [38]. The KT muscle application on the quadriceps was used. Proprioception, balance, and functional performance were assessed after KT application using the Lysholm scale, anteroposterior shift of the tibia, active angle reproduction test, modified star excursion balance test, and single-hop distance. They concluded that KT has benefits in people with ACL rupture but cannot fully compensate for functional deficits. KT could be used to assist knee strengthening during rehabilitation.

There are also studies available in the literature concerning the assessment of the stability of the knee joint after ACL reconstruction. According Aghdam et al. the standing stability after ACL reconstruction decreased significantly, which may be due to the effects of the surgery on sensory mechanism of ACL and inability of patients to return to their previous deep sense perception and knee proprioception [39]. On the other hand, Ogrodzka-Ciechanowicz et al. claim that after the ACL reconstruction and individual rehabilitation the static stability of the knee improves [40].

Therefore, the results of own research make an important contribution to the analysis of the effectiveness of the KT ligamentous technique after ACL rupture. It can be concluded that application of KT reduces the value of sway at the CoP in patients with ACL rupture. This in turn results in better static stability of the joint and body posture. The obtained results indicate the effectiveness of KT in improving the static stability of the knee after ACL rupture. The application, which was evaluated, directly affects the standing stability of the knee. It maintains a proper dorsal glide, thus blocking the giving-way syndrome, which is the cause of significant movement limitations of patients after the ACL rupture.

The beneficial effect of the KT is explained by its impact on the axial positioning of joints, which improves their stability.

While selecting the appropriate therapy for the patient after ACL rupture one should keep in mind that KT can be part of it, but should not be used as a single method in order to achieve best results.

The stability of the knee joint results from several factors influencing one other, such as anatomy of a joint, body weight and loads acting on the joint. On the one hand, bone structure does not provide joint stability. However, ligaments, articular capsule and other soft tissues surrounding the joint are important in maintaining proper joint control. During physical activity pressure forces acting on a joint (resulting from body weight and activity of muscles) protect it additionally [41].

In the case of ACL injury, the stabilizing function can be taken over by other articular structures, but this requires appropriate training. By adding the KT method into therapy, which according to its assumptions is to perform a stabilizing function, you can expect an improvement in static control of body posture.

The conducted own research indicates the need for the continuation of the undertaken issue. A growing number of patients after ACL rupture and their increasing expectations regarding the effectiveness of treatment incline to broadly analyse of the problem. The diagnosis should include all indicators that may contribute to an increase in a patient’s satisfaction, but also to minimise injury complications. In assessing the effectiveness of therapy, it is therefore purposeful to use stabilometric platforms which should become the standard in the diagnosis of patients after ACL rupture.

The limitation of the conducted research was the small number of comparable studies. This was the first study using the CQStab2P stabilographic platform to assess the effectiveness of KT among people with ACL rupture. This impeded the discussions on the data, but at the same time reflected the unique design of the study. Further research is needed on the effectiveness of KT in the treatment of patients with ACL rupture based on postural control assessment to determine clinical outcomes and allow discussion of its clinical relevance.

Summarising the above results, it should be concluded that after applying KT there is reduced sway of the CoP, which means the patient has more control over standing. The results obtained are conducive to the effectiveness of application of the selected KT technique in patients after ACL rupture. It seems, however, that the issues related to KT and its performance or lack of influence on the improvement of human body movement requires further research, taking into account other aspects of the problem studied. The more that there are few reports in the literature evaluating the effectiveness of KT in the context of improving static stability.

## Conclusions

1. Application of KT in patients after ACL rupture shortened the total path length and improved the value of parameters in the frontal and sagittal planes in experimental group, which may suggest the potentially greater improvement in these parameters.
2. KT application decreased CoP displacement velocity values in particular axes, which means increased improvement in the control of standing position.
3. By improving the values of the analyzed variables, the KT application is able to compensate for the loss of static stability of the knee.

### Practical Implications

1. Application of KT in the treatment of patients with a complete ACL rupture has beneficial early effect on achieving static stability, assuming that this is a method supporting the treatment of patients with ACL rupture.
2. KT method could be a therapy offered for patients preparing for surgery of ACL reconstruction.
3. KT method should be prescribed for patients with musculoskeletal injuries, as suggested by the creators of KT.

## Data Availability

The datasets used and/or analysed during the current study are available from the corresponding author on reasonable request.

## Abbreviations

ACL: Anterior Cruciate Ligament
CoP: Centre Of Pressure of the Foot
KT: Kinesiotaping
SP: Total path length, on both axes of rectangular coordinate Y0X [mm]
SPAP: Statokinesiogram path length on the Y axis [mm]
SPML: Statokinesiogram path length on the X axis [mm]
